# The Surprises of General Practice

**Published:** 1981

**Authors:** Philip Radford

**Affiliations:** General Practitioner, Westbury-on-Trym, Bristol


### The Surprises of General Practice

**Philip Radford**
General Practitioner, Westbury-on-Trym, Bristol

It was not until I entered general practice that I realised how difficult it was to plan the work of a given day in that occupation. Domiciliary midwifery took a lot of time and an expected date of delivery could only be an approximation; and influenza epidemic made life a chaotic rush and even outbreaks of measles or chicken pox made the visiting list longer than one would have wished, coupled as it usually was with an increase in late evening and night work. Yet one of the attractions of general practice is the element of surprise which it brings: the unforeseen event in a day of busy routine can make that day memorable. For this reason, medical practice is so rewarding and set apart from most other occupations where the toil of the day can be accurately predicted.

As an example, a father telephones me one evening asking for a visit to his schoolgirl daughter who was in bed with backache. Expecting to find a urinary tract infection as the cause of the problem, I noticed that the patient was lying on her side and insisting on staying there. Mother was out for the evening, father had discreetly disappeared and little history of the complaint could be obtained. Anyway, the patient was adamant that examination was quite unnecessary and that all she wanted was an injection ('a shot') for the pain; in her opinion, a doctor should be able to treat backache without examining any part of the body except (as a real concession) the back. Of course, the girl was nearly at full dilatation of the cervix and a healthy infant was born 2-3 hours later.

In my experience, a father of a girl with an unexpected pregnancy is either very retiring and reticent or else he is rather aggressive. A somewhat belligerent father appeared in the bedroom as I confirmed that his 19-year-old student daughter was in advanced (illegitimate) pregnancy; his assertion was that student grants, that is taxpayers' and his money, are awarded for purposes other than those of human proliferation. One year later, almost to the day, the same father was remonstrating with his daughter in the same bedroom; this time the girl, still unwedded, was having a spontaneous abortion of a second pregnancy.

A young lady of the same age was involved when her boyfriend who she was visiting for the weekend, telephoned early one morning saying that the girl was feeling faint, had pain in the abdomen and was passing her urine frequently. I concluded, after examining the patient, that she was suffering from a urinary tract infection and took away a specimen for bacterial culture. A few hours later, to my surprise, I was told she was feeling worse. I found the patient pale with a rapid pulse and right shoulder-tip pain, but there was no evidence of an ectopic gestation; at laparotomy, it was discovered that she was bleeding at the site of a ruptured ovarian cyst. Presumably the early intra-peritoneal bleeding had irritated the bladder but, clearly, I should have paid more attention to the feelings of dizziness and faintness. Certainly, the eventual diagnosis was an unexpected one for me.

Then I once diagnosed cystitis in an elderly woman with marked frequency of micturition and pyrexia. As abdominal pain increased an exploratory operation was carried out, with the finding of an inflamed appendix firmly adherent to the bladder. Both pelvic surgery and gynaecology may bring surprises for the doctor and for the patient as well: it is remarkable how often vaginal discharge can be cured by the removal of a forgotten vaginal tampon. I have been surprised too by observing on routine examination that some women with profuse pubic hair appear to go to great trouble to part and comb this hair; occasionally, one has the impression that certain individuals try to style it as well.

Also an unexpected finding, when I started practice, was the frequency with which the chests of coughing or influenzal patients were annointed with goose grease or other more up-to-date and costly oily rubs. With my zeal for palpation and percussion, I found that much time had to be expended in trying to soap off that fatty film, not only from my hands but from my pen and stethescope as well. Soon I learned to smell out the affected patients who tended to receive a rapid prescription rather than a detailed examination. In connection with odour, I am reminded of the old country custom of applying the lungs of lambs to the feet of pneumonia sufferers when, after a few days, the doctor could obviously smell out those households which had little faith in his therapeutic ability.

Sometimes patients have too great a belief in the doctor's power of healing. I recall a man who insisted that I gave him an ointment to cure a cystic lump of the scalp. He did not believe me when I said he could only be helped by surgical removal but, in due time, he agreed to be placed on a hospital waiting list. After a delay of several months the cyst was removed but my faith in a surgical cure was altered when histology showed the lump to be a reticulo-sarcoma. Similarly, most doctors have been asked at some time to prescribe an ointment for a sore on the breast, only to find, on inspection, a fungating and inoperable carcinoma. Perhaps doctors should be careful of making a diagnosis of any lump, however, before histological examination has been performed. As an example, a mid-line neck lump in a young male which, to my mind, was a thyroglossal cyst, was proved to be caused by Hodgkin's disease.

Surprise in general practice is not always associated with medical matters and observations on many subjects, natural history, for instance, may be made in the course of one's work. Many times, when out on night visits, I have seen foxes crossing the road in the glare of headlamps; once a fox ran for several metres along the top of a wall, moving parallel with me and another animal disputed face-to-face with a cat while I watched from my stationary car. Again, a parson naturalist, after his cardiac assessment, gave me unexpected pleasure one rainy June day by showing me a young cuckoo in a dunnock's nest in his garden. The cuckoo pushed firmly as I place a finger tip on its lower back, thus illustrating how it had expelled the newly-hatched young dunnocks, the rightful occupants, from the nest. Later that day, I felt a similar sensation on my examining finger, when it was pressed on by an advancing foetal head.

Taking a history may give rise to an unexpected story, not always of strict medical relevance but connected, perhaps, with particular social conditions. Thus, an elderly patient, when referring to her childhood, mentioned how her elder brother had sent her to a house to ask for the boots of a recently dead boy and how thankful she had been when the boots were handed over. Another patient recounted how she had been ordered to beg a shoe box to act as a coffin for her mother's still-born and premature foetus. Social and family life in Britain in the poorer groups of the community was remarkably tough yet pragmatic and this situation is, after all, only going back a few decades. Equally, one senses great kindness and help for neighbours in these rather impoverished areas; I noticed this constantly when working in Bristol although a change in attitude came with the building of new housing estates and multistorey flats.

While I have noted unexpected aid and support in time of need amongst all classes of society, at times I have been amazed at the lack of consideration and care shown by some individuals. I recall seemingly most responsible people who, when asked to look after an aged parent with a relatively minor illness, have refused, saying that it is up to the doctor to find a hospital bed. Occasionally, under such circumstances, help may come from the local clergy if they are close to the family and happen to be already involved in the problem. There are still people who would not like their vicar to think that they would decline help to an ailing relative, although they do not mind stating this to the doctor.

Only rarely have I known a clergyman to be reluctant to visit an ill member of his flock when asked to do so. But I do remember a bachelor parson who said that my patient had a very bad reputation (which was true) and he did not think that members of his congregation would approve of him associating with her. Rather impudently, I suggested that '... joy shall be in heaven over one sinner that repenteth ...' and as this happened to be the text of the clergyman's next sermon he agreed to visit as soon as possible. He was, naturally, spotted coming from the house but as the visit gave real psychological benefit, I trust he was consoled.

Another minister of religion, whose ill wife I was seeing regularly, was a young man who was due to preach at a chapel many miles away during a period of heavy snow. On the Monday after the sermon the minister told me that he had had to walk to the service as the roads were impassable for motor traffic; moreover, his congregation had consisted of two people, including the organist. I asked him if the service had been worth the effort of walking there and he replied that no hardship was too great if two people were to hear God's word and its meaning. Feeling quite humbled, I regretted that I had asked the question but as the minister's wife had nearly recovered from her illness I have no doubt that the minister was grateful that his prayers had been answered.

I also felt humbled when I saw a 16-year-old girl at her home as I had only sent her into hospital 24 hours previously with a confident diagnosis of acute appendicitis. Her mother told me, reproachfully, that the surgeon had said that the pain came from the ovary and I was handed a hospital note to this effect. Nevertheless, I failed to understand how ovarian pain could arise at this age only a day or two after the end of menstruation, so I was not really surprised when the girl's condition deteriorated a few hours later. I thought it unfortunate that when the patient was re-admitted, for immediate appendicectomy this time, she was under the care of a different surgical team; admittedly, I have made many clinical errors but, when they have occurred, I have tried to learn from them. It must be remembered that general practitioners, unlike hospital doctors, must continue to live and work amongst their patients, whatever mistakes have occurred.

Just as practitioners accept the surprise of emergency surgery, so they accept the unpredictability of the outcome of many psychiatric emergencies. To take an example, I recollect one colourful and verbose middle-aged lady who lived quite happily in squalor in a once pleasant detached house. Regretfully, she started to shout abuse at local residents, especially the children; she neglected to feed herself, her clothes became more dirty and increasingly unconventional and she refused to pay any bills. I visited her in the company of a consultant psychiatrist, after pushing through the bushes of a garden unattended for many years and stepping over innumerable milk bottles. Not surprisingly, we failed to persuade this disturbed patient to enter a mental hospital on a voluntary basis. Yet the surprise came a week later, when I was asked to give my patient a reference as she had applied to become the matron of a large local authority children's home!

Obviously the general practitioner's job can never be one of monotonous routine. Although the work is demanding and, in my opinion, requires continuity to be of real value for the patient, it is one of the most vital as well as one of the most important of occupations. To my mind, no one should become a general practitioner who cannot keep a sense of humour and who does not enjoy the unexpected turn of event; without doubt, frustrations and problems, many of them insoluble, are the essence of general practice. Surely, it is the factor of surprise which gives such fascination to the family doctor's work and I sometimes think that, in consequence, the practitioner is given training in dealing with almost any known situation.

